# The effects of nicotine use during adolescence and young adulthood on gray matter cerebral blood flow estimates

**DOI:** 10.1007/s11682-023-00810-5

**Published:** 2023-10-18

**Authors:** Kelly E. Courtney, Rachel Baca, Courtney Thompson, Gianna Andrade, Neal Doran, Aaron Jacobson, Thomas T. Liu, Joanna Jacobus

**Affiliations:** 1 Department of Psychiatry, University of California San Diego, 9500 Gilman Drive, MC 0405, La Jolla, CA 92093 USA; 2 Veterans Affairs San Diego Healthcare System, La Jolla, CA USA; 3 Department of Radiology, University of California San Diego, La Jolla, CA USA

**Keywords:** Cerebral blood flow, Gray matter, Nicotine, Cotinine, adolescence, Young adults

## Abstract

Nicotine and tobacco product (NTP) use remains prevalent in adolescence/young adulthood. The effects of NTPs on markers of brain health during this vulnerable neurodevelopmental period remain largely unknown. This report investigates associations between NTP use and gray matter cerebral blood flow (CBF) in adolescents/young adults. Adolescent/young adult (16–22 years-old) nicotine users (NTP; N = 99; 40 women) and non-users (non-NTP; N = 95; 56 women) underwent neuroimaging sessions including anatomical and optimized pseudo-continuous arterial spin labeling scans. Groups were compared on whole-brain gray matter CBF estimates and their relation to age and sex at birth. Follow-up analyses assessed correlations between identified CBF clusters and NTP recency and dependence measures. Controlling for age and sex, the NTP vs. non-NTP contrast revealed a single cluster that survived thresholding which included portions of bilateral precuneus (voxel-wise alpha < 0.001, cluster-wise alpha < 0.05; ≥7 contiguous voxels). An interaction between NTP group contrast and age was observed in two clusters including regions of the left posterior cingulate (PCC)/lingual gyrus and right anterior cingulate cortex (ACC): non-NTP exhibited positive correlations between CBF and age in these clusters, whereas NTP exhibited negative correlations between CBF and age. Lower CBF from these three clusters correlated with urine cotinine (*r*s=-0.21 – − 0.16; *p*s < 0.04) and nicotine dependence severity (*r*s=-0.16 – − 0.13; *p*s < 0.07). This is the first investigation of gray matter CBF in adolescent/young adult users of NTPs. The results are consistent with literature on adults showing age- and nicotine-related declines in CBF and identify the precuneus/PCC and ACC as potential key regions subserving the development of nicotine dependence.

## Introduction

Despite decades of public health efforts, use of nicotine and tobacco products (NTPs) remains high among adolescents and young adults in the US. Recent estimates indicate past-month prevalence of 20.7% for e-cigarettes and 4% for combustible cigarettes among 12th graders (Miech et al., 2023), and 14.1% for e-cigarettes and 11.5% for combustible cigarettes among young adults (Center for Behavioral Health Statistics and Quality, 2022). Alarmingly, since 2019, age of initiation of e-cigarette vaping has continued to decrease, and use severity (levels of addiction and intensity of use) of current e-cigarette users has continued to increase (Glantz et al., 2022). Use of e-cigarettes before age 18 is associated with a three-fold greater likelihood of becoming a regular user of combustible cigarettes by young adulthood (Pierce et al., 2021), which increases risk for nicotine dependence across the lifespan and for problematic use of other substances (Hindocha et al., 2020; Hindocha & McClure, 2020). Thus, NTP use remains a key concern in late adolescence with well-established risks for later addiction.

Extensive dynamic morphological and cellular brain changes occur throughout adolescence and the early 20s, such as an increase in neural circuitry specialization and interconnectedness (Giedd et al., 2015; Gogtay et al., 2004), which may be altered by use of psychoactive substances such as nicotine. Nicotine use during adolescence is associated with worsened performance on measures of working memory and attention (Jacobsen et al., 2005; Treur et al., 2015). Brain structural abnormalities, as indexed by magnetic resonance imaging (MRI) scans, have been associated with nicotine use in younger populations, including increased right caudate volume and reduced cortical thickness in frontal, insula, parahippocampal, and temporal regions (Li et al., 2015), and smaller left amygdala and thalamus volumes (Hanlon et al., 2016), relative to controls. Further, longitudinal studies have observed differential cortical morphometry trajectories, particularly in the frontal cortices, dependent on tobacco use during adolescence (Akkermans et al., 2017; Chaarani et al., 2019a). The effects of nicotine use on brain health markers may also be moderated by biological sex, as a recent report found smaller left caudate volumes in young adult male tobacco smokers as compared to male non-smokers, yet no differences were observed between female smokers and non-smokers (Lin et al., 2020).

Evaluation of cerebral blood flow (CBF) via arterial spin labeling (ASL) or positron emission tomography represents an alternative technique to index brain health. CBF is closely associated with glucose metabolism (Jueptner & Weiller, 1995) and brain function (Raichle et al., 1976). CBF may be particularly relevant to the study of adolescent brain health as it supplies oxygen and energy substrates throughout the brain that are critical for effective neurodevelopment (Fantini et al., 2016). CBF estimates evince several nonlinear age-by-sex interactions from childhood into late adolescence (Satterthwaite et al., 2014; Taki et al., 2011), whereby CBF generally increases across childhood (Paniukov et al., 2020), declines into early puberty, but then diverges post-puberty with females showing an increase, and males continuing to show a decrease, into young adulthood (Satterthwaite et al., 2014).

In adults, chronic/heavy tobacco smoking is associated with a decline in CBF (Kubota et al., 1983; Vafaee et al., 2015); yet, the acute effects of nicotine are mixed and seem to support region-specific decreases and/or increases (Domino et al., 2000; Rose et al., 2003; Wennmalm, 1982). Sex differences have also been observed in CBF estimates of adults with nicotine dependence. Specifically, sated adult nicotine-dependent female cigarette smokers evinced stronger CBF functional connectivity between hippocampus/amygdala seed regions and the bilateral anterior insula, rostral anterior cingulate cortex, and inferior parietal lobule contrasted to comparable male smokers (Wetherill et al., 2014). The effects of nicotine on CBF in younger populations remains largely untested outside of our own work, which observed an interactive effect of nicotine and cannabis use history on white matter CBF in late adolescence (Courtney et al., 2020). To our knowledge, the effects of nicotine on gray matter CBF in adolescents/young adults remains unknown. Given the specified age-related trajectories of CBF in healthy adolescents, it is imperative to evaluate substance-related effects on CBF during this vulnerable period of rapid neurodevelopment.

Therefore, this study investigated associations between recent NTP use and gray matter CBF in a sample of adolescent/young adults (aged 16–22). It was hypothesized that NTP users would evince lower overall CBF estimates compared to controls; region-specific hypotheses were not made due to a lack of consilience in the literature. Given the findings related to age- and sex-specific effects on CBF, we directly tested the role of these variables in the nicotine group analysis.

## Methods

### Participants & procedures

Data were derived from a study on the structural and functional neural effects of nicotine and cannabis co-use in adolescence/young adulthood. As previously reported (Courtney et al., 2020, 2022), participants (N = 225; ages 16–22) were recruited via flyers posted physically and electronically at high schools, community colleges, four-year universities, and social media sites targeting San Diego County. Initial recruitment was stratified based on use of NTP, cannabis products, or both during the previous 6-months (Courtney et al., 2020). For this study, participants were recategorized based on NTP frequency only: the NTP group endorsing > = 26 NTP use episodes in the previous 6-months (~ at least weekly) and the non-NTP group endorsing < 26 NTP use episodes in the previous 6-months. NTP use was defined as the use of any combination of electronic cigarettes (e.g., vape pens, e-hookah), combustible cigarettes, hookah with tobacco, tobacco pipe, cigars (including blunts, spliffs), snus, smokeless tobacco, chew, snuff, and/or nicotine replacement.

Exclusions included > 10 lifetime illicit substance uses, lifetime DSM-5 psychiatric disorder other than tobacco and/or cannabis use disorder, acute influence of cannabis or alcohol use at study visit (confirmed with urine, breathalyzer, and oral fluid toxicology), use of any psychoactive medications, major medical issues, MRI contraindications (e.g., metal braces, implanted metal, etc.), or history of prenatal substance exposure or developmental disability.

Participants provided written informed consent in accordance with the University of California, San Diego Human Research Protections Program prior to participating in a single, four-hour visit in the laboratory. Participants then underwent a battery of interviews and self-report assessments covering demography, mental health, substance use, and neurocognitive functioning, followed by a magnetic resonance imaging session. Participants were asked to abstain from cannabis and alcohol use within 12 h prior to the appointment – oral fluid, urine, and breathalyzer for alcohol corroborated self-reported substance use. Urine samples were used to quantify cotinine (nicotine’s major metabolite) for nicotine users and to confirm abstinence from illicit substances (quantification conducted by Redwood Toxicology). Participants abstained from caffeine intake for at least 30 min prior to scanning. They were not required to abstain from NTP use to avoid nicotine withdrawal effects during testing and time of last use was documented (range: 0.03–1460 h, NTP median = 24 h, non-NTP median = 624 h (n = 52); see Table 1).

**Table 1:** Sample demographics and nicotine and tobacco product (NTP) use by group

Variable	Group [mean (standard deviation) or %]		*t*(df) or *χ²*	*p-*value
	Non-NTP	NTP		
Total N	95	99		
Age	19.08 (1.67) [*16–22*]	19.77 (1.47) [*16–22*]	-3.03(192)	0.003
% Male	41.0%	59.6%	6.67	0.01
% White	50.5%	53.5%	0.18	0.68
% Hispanic	41.0%	33.3%	1.24	0.27
Education Years Completed	12.79 (1.57) [9–16]	13.21 (1.37) [10–16]	-2.00(192)	0.05
Age of Onset of Nicotine Use	16.81 (1.73) [*13–21*] n = 52	16.17 (2.08) [8*–20*]	1.88(149)	0.06
Years of Nicotine Use	2.65 (2.20) [*0–9.00*] n = 52	3.60 (2.15) [*0–12.00*]	-2.54(149)	0.006
Days Since Last Nicotine Use	134.86 (297.47) [*2-1460*] n = 52	4.25 (9.33) [*0–70*]	4.38(149)	< 0.001
Nicotine Use Episodes Previous 6 Months	3.76 (6.07) [*0–25*]	1613.99 (3126.12) [*28-23275*]	-5.02(192)	< 0.001
Nicotine Dependence (WISDM PDM)	1.04 (0.32) [*1.00-4.13*]	2.95 (1.73) [*1.00-6.94*]	-10.54(192)	< 0.001
Nicotine Craving (WISDM Craving)	1.06 (0.45) [*1.00-5.25*]	2.89 (1.84) [*1.00–7.00*]	-9.40(192)	< 0.001
Urine Cotinine (ng/mL)	1.23 (7.25) [*0–52*] n = 57	268.02 (220.27) [*0-500*] n = 86	-9.13(141)	< 0.001

Note: WISDM PDM = Brief Wisconsin Inventory of Smoking Dependence Motives Primary Dependence Motives scale; WISDM Craving = Brief Wisconsin Inventory of Smoking Dependence Motives Craving subscale

### Measures

Demographic data (e.g., age, sex at birth, race/ethnicity, education) were derived from a demographic and psychosocial interview. The Customary Drinking and Drug Use Record structured interview (Brown et al., 1998), modified to include additional nicotine and cannabis questions (Jacobus et al., 2018; Karoly et al., 2019a; Karoly et al., 2019b), was used to assess lifetime history of substance use and substance-related problems. The Brief Wisconsin Inventory of Smoking Dependence Motives (Brief WISDM; (Smith et al., 2010), modified to include all NTPs, was used to generate scores on the Primary Dependence Motives (PDM) scale and Craving subscale– reflective of severity of nicotine dependence and craving, respectively. For the purposes of this report, current frequent nicotine use was defined as at least weekly NTP use, on average, during the previous 6 months.

### Imaging acquisition and processing

#### MRI specifications

Participants were scanned on a 3.0 Tesla GE Discovery MR750 scanner with a 32-channel receive head coil at the UCSD Center for Functional MRI. A high-resolution T1-weighted anatomical fast spoiled gradient echo (FSPGR) scan was acquired with TI/TE/TR = 1060/2/2500ms, 256 × 256 matrix, flip angle = 8˚, FOV = 256 mm, 1.0 mm^3^ voxels.

#### Optimized pseudo-continuous arterial spin labeling (OptPCASL) specifications and processing

As previously described (Courtney et al., 2020), resting CBF was measured using an OptPCASL method developed at UCSD (Shin et al., 2012) that provides a high level of robustness and sensitivity to make time-efficient measurements in gray and white matter tissue (Lu et al., 2009). The protocol consisted of an OptPCASL scan (scan time 9:27 min, 64 × 64 matrix, FOV = 240 mm, 24 axial contiguous slices, 6 mm thick, single-shot spiral acquisition with TE = 3.2ms; TR = 4500ms, tag duration = 1900ms, post labeling delay = 1900ms) with background suppression and additional calibration (for CBF quantification) and field maps scans (Shin et al., 2012). The use of the short echo time spiral acquisition minimizes susceptibility-related dropouts. Field map information was used during image reconstruction to correct for susceptibility-induced distortions (Noll et al., 2005).

The analysis pipelines found in Shin et al. (2016;, 2013) were used to implement distortion correction and conversion of raw OptPCASL data to quantitative CBF maps. These pipelines utilize relevant tools from in-house MATLAB scripts and multiple neuroimaging software packages (AFNI, Cox, 1996; Freesurfer, Dale et al., 1999; FSL, Smith et al., 2004). A mean ASL image was formed from the average difference of the control and tag images. This image was then corrected for coil inhomogeneities and converted to absolute units of CBF (mL/100 g^-1^ min^-1^) using proton density weighted images acquired 30 s after the OptPCASL sequence.

Consistent with previous reports (Courtney et al., 2020), the CBF and aligned high resolution anatomical images were warped to Talairach space using AFNI’s auto_tlrc function and resampled to a 4 × 4 × 4 mm resolution grid using AFNI’s adwarp. Voxels with negative intensities were replaced with zero (Brown et al., 2003). All data were visually screened for quality and alignment. Thirty-one subjects were excluded due to incomplete data, technical issues, or excessive movement during scanning. An average anatomical image was created across all participants with usable gray matter ASL data (N = 194) and segmented using FSL’s FAST algorithm to define cerebral spinal fluid, gray matter, and white matter regions. Cerebellar values were omitted from the analysis by masking in the atlas space due to inconsistencies in coverage during acquisition (Alsop et al., 2008; Liu et al., 2011). The gray matter segmentation (with cerebellum removed) was used as a mask for each participant CBF image in the statistical analyses.

### Statistical analysis

Nicotine use group differences on demographic variables were explored by independent *t-* or *χ²* tests (significance threshold set at *p* < .05). Consistent with methods previously used by the authors (Courtney et al., 2019, 2020), NTP and non-NTP group perfusion estimates were contrasted using AFNI 3dttest++. Age and sex at birth were entered in the same model as covariates of interest given the observed group differences in these theoretically relevant variables. AFNI’s Clustsim nonparametric randomization/permutation option with a voxel-wise alpha of 0.001 and cluster-wise alpha of 0.05 was used to determine the cluster size threshold (Cox et al., 2017; Eklund et al., 2016), resulting in an estimated cluster size threshold of 7 contiguous voxels. The Talairach Daemon atlas was used to assist in region identification (Lancaster et al., 1997).

The single cluster identified from the main effect of nicotine use group contrast (non-NTP vs. NTP) controlling for age and sex, as well as the two clusters from the nicotine group x age interaction effect controlling for sex, that surpassed thresholding were used to create cluster-specific functional masks. These cluster masks were then applied to the CBF images and the mean CBF estimates were extracted from each cluster for each participant individually. A priori correlations between CBF estimates from each cluster and urine cotinine level, nicotine dependence severity (from the Brief WISDM – PDM scale), and self-reported NTP recency (days since last use) were tested via bivariate Pearson correlation tests (three correlations per region; p-value threshold Bonferroni corrected at 0.05/3 = 0.017). Exploratory correlations between CBF estimates from each cluster and nicotine craving (from the Brief WISDM – Craving subscale) were also conducted to determine specificity of the effect.

## Results

### Participants

The final sample (N = 194) was roughly split on sex at birth (50.5% male) and 52.1% self-identified as White (see Table 1). The non-NTP and NTP groups were found to differ significantly on sex, age, and as expected, on all nicotine-related metrics except age of onset of nicotine use (*p*s < 0.05). Within the NTP group, 80.9% endorsed greater vaping, versus smoking of combustible products, as their primary nicotine delivery method. Although the NTP group endorsed approximately 9 NTP use episodes per day if averaged across the 6 months, their reported NTP use was highly episodic in nature and may not be best captured by a daily average.

### Whole-brain gray matter CBF results

**Fig. 1:**
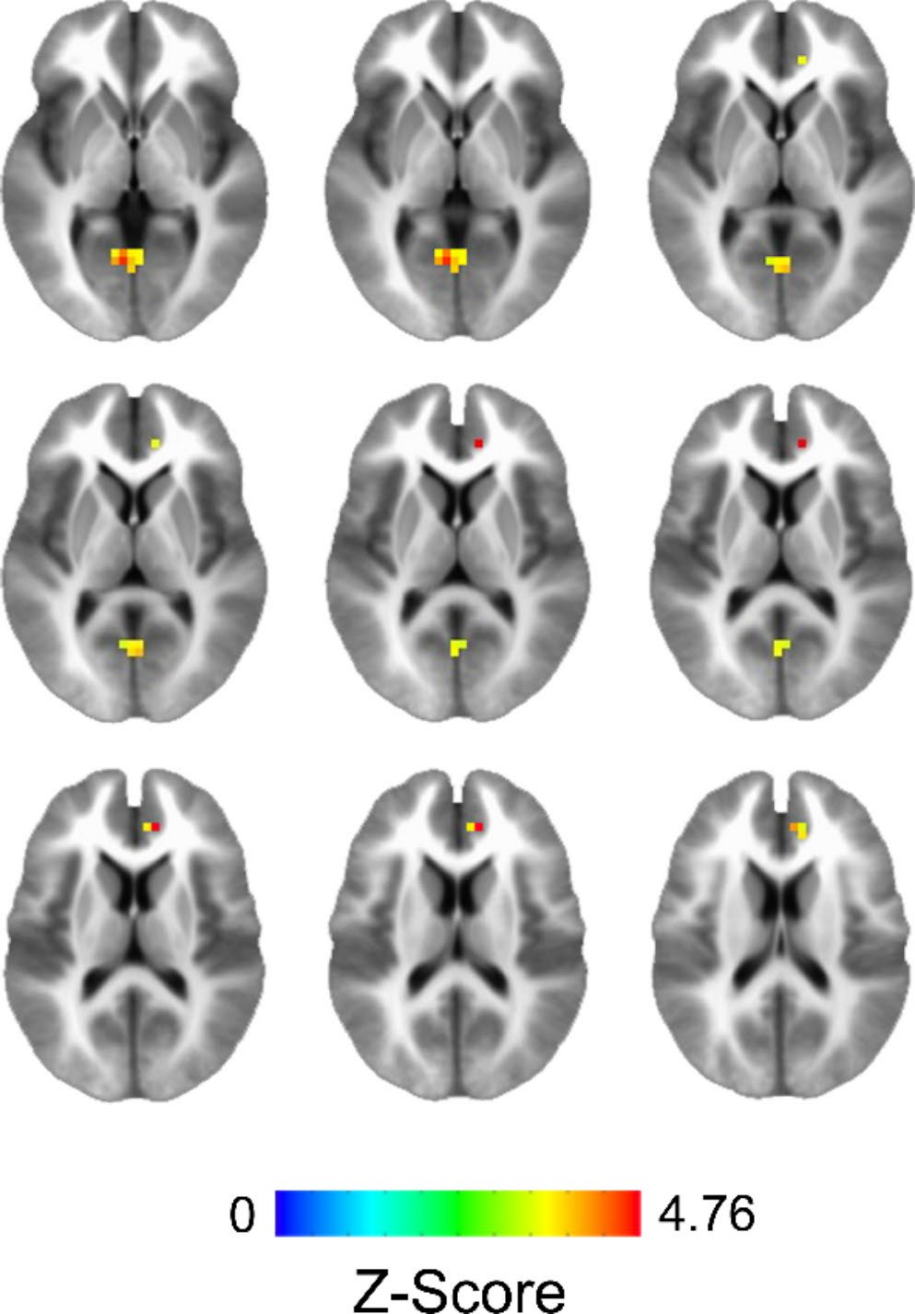
Clusters of significant cerebral blood flow (CBF) response obtained from the interaction effect between nicotine use group (non-NTP vs. NTP) and age, controlling for sex at birth (thresholded at a voxel-wise alpha of 0.001, cluster-wise alpha of 0.05; ≥ 7 contiguous voxels). The images are shown in radiological convention where the left hemisphere is displayed on the right side of the image

**Fig. 2:**
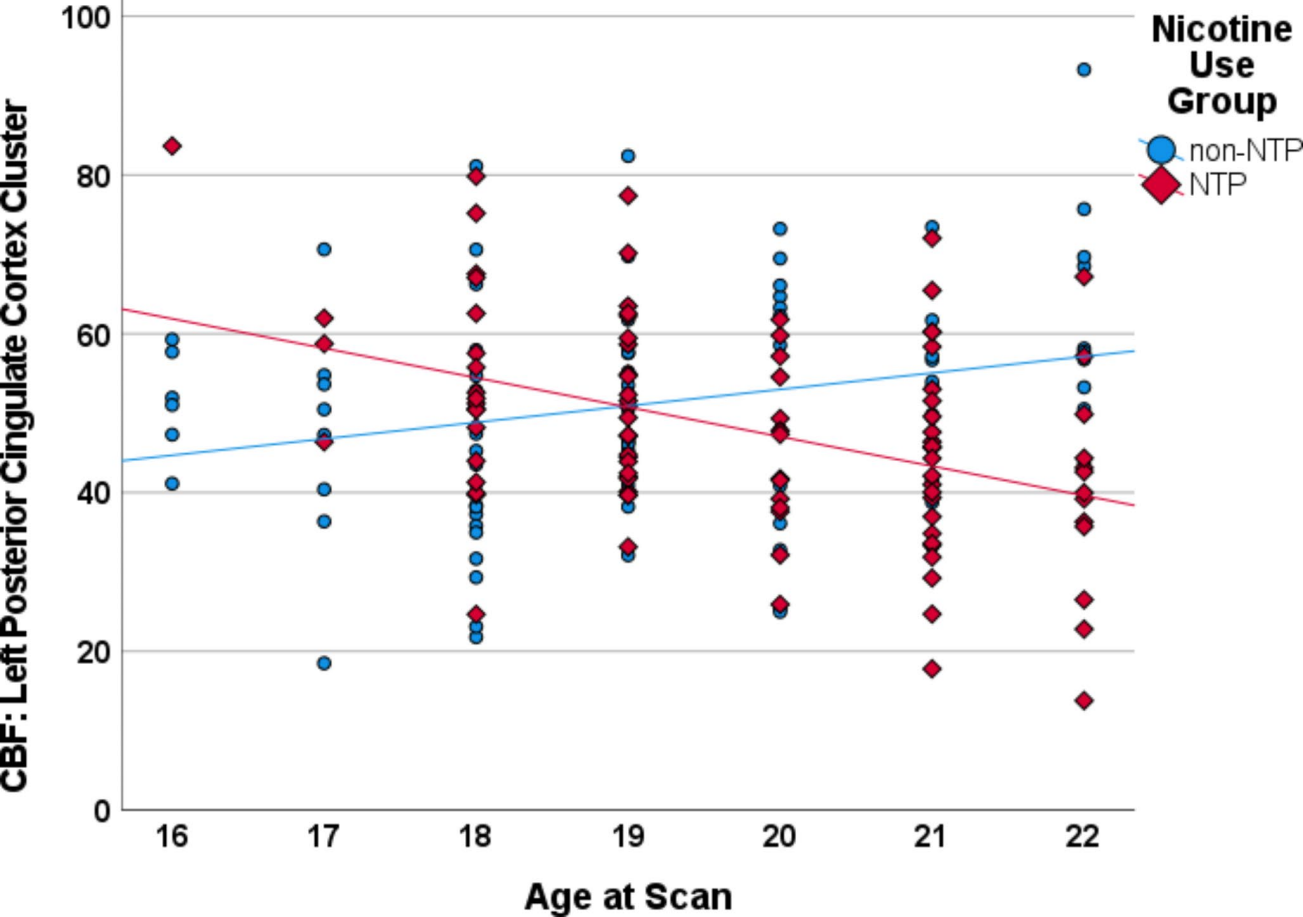
Scatterplot depicting the correlations between age and cerebral blood flow (CBF) extracted from the left posterior cingulate cortex (PCC)/lingual gyrus cluster for the nicotine (NTP) and non-nicotine groups (non-NTP), controlling for sex at birth. Scatterplot presented for visualization purposes only - no statistics were calculated to avoid potential inflation of the correlation estimate

**Fig. 3:**
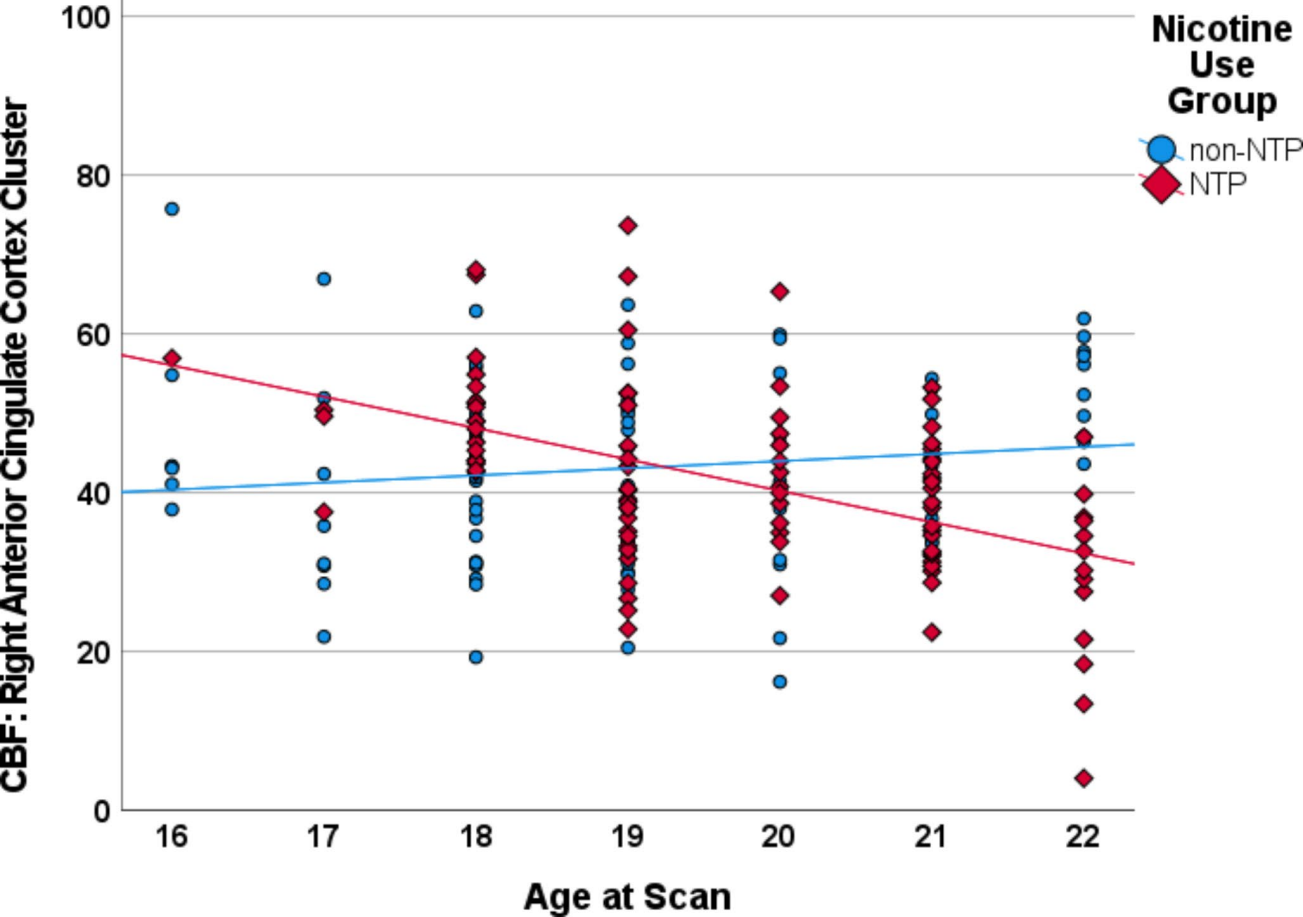
Scatterplot depicting the correlations between age and cerebral blood flow (CBF) extracted from the right anterior cingulate cortex (ACC) cluster for the nicotine (NTP) and non-nicotine groups (non-NTP), controlling for sex at birth. Scatterplot presented for visualization purposes only - no statistics were calculated to avoid potential inflation of the correlation estimate

Analysis of the whole-brain gray matter contrast between non-NTP and NTP group perfusion estimates (non-NTP vs. NTP) controlling for age and sex at birth revealed a single positive cluster that survived thresholding with a peak voxel located in the left precuneus and extending to the right precuneus (see Table 2). Examination of the interaction between nicotine use group (non-NTP vs. NTP) and age revealed two clusters of significant perfusion estimates with peak voxels located in the left posterior cingulate cortex (PCC)/lingual gyrus and right anterior cingulate cortex (ACC; see Table 2 and Fig. 1): non-NTP exhibited positive correlations between CBF and age in these clusters, whereas NTP exhibited negative correlations between CBF and age (Fig. 2 and Fig. 3). No significant interaction between nicotine use group (non-NTP vs. NTP) and sex at birth was observed.

**Table 2:** Cluster of significant cerebral blood flow (CBF) response from the main effect comparing nicotine use groups (non-NTP vs. NTP; controlling for age and sex at birth) and from the interaction effect between nicotine use groups (non-NTP vs. NTP) and age, controlling for sex at birth (thresholded at a voxel-wise alpha of 0.001, cluster-wise alpha of 0.05; ≥ 7 contiguous voxels)

Region (peak)	Cluster Voxels	Talairach Coordinates			Z-Score	Alpha
		X	Y	Z		
**Main effect of nicotine use group:**						
Cluster 1: Left precuneus – extending to right precuneus	8	2.0	69.0	16.0	3.63	< 0.04
**Interaction of nicotine use group X age**:						
Cluster 1: Left posterior cingulate/lingual gyrus	22	2.0	61.0	4.0	4.58	< 0.01
Cluster 2: Right anterior cingulate cortex	7	-10.0	-39.0	16.0	4.76	< 0.05

### A priori CBF cluster correlations with nicotine use and dependence

Across the sample, CBF extracted from the main effect of nicotine group cluster controlling for age and sex (bilateral precuneus) negatively correlated with urine cotinine (r=-.17, *p* = .04), and to a lesser extent nicotine dependence severity (r=-.13, *p* = .07), but not NTP recency (r = .08, *p* = .33). CBF extracted from the first cluster identified from the interaction with age controlling for sex (left PCC) negatively correlated with urine cotinine (r=-.21, *p* = .01) and nicotine dependence severity (r=-.16, *p* = .02), but not NTP recency (r = .01, *p* = .89). CBF extracted from the second cluster identified from the interaction with age controlling for sex (right ACC) negatively correlated with nicotine dependence severity (r=-.15, *p* = .03), and to a lesser extent urine cotinine (r=-.16, *p* = .06), but not NTP recency (r = .02, *p =* .82). Only the correlation between left PCC CBF (identified from the interaction with age) and urine cotinine level survived Bonferroni correction (*p* < .017). The follow-up correlations between CBF estimates and craving scores revealed results largely consistent with the nicotine dependence severity correlations (main effect- bilateral precuneus: r=-.11, *p* = .11; interaction- left PCC: r=-.16, *p* = .03; interaction- right ACC: r=-.15, *p* = .04).

## Discussion

This report sought to examine associations between nicotine use and gray matter CBF markers in a sample of late adolescents/young adults. The results suggest NTP use may moderate the normal age-dependent changes in gray matter CBF, where CBF in the left PCC and right ACC appears to decline in older adolescents/young adults endorsing frequent NTP use compared to an observed incline in CBF estimates in these regions in similarly aged non-users. Further, patterns of correlations between PCC and ACC CBF and measures of current nicotine use/dependence support the findings that greater NTP use and dependence are associated with diminished brain health during this prolonged period of neurodevelopment.

Acute nicotine administration appears to induce a dose-dependent vasodilation effect on cerebrovasculature (Iida et al., 1998; Toda, 1975), yet the effects of chronic nicotine exposure on cerebrovasculature remain somewhat ambiguous. Generally, reports suggest a decline in CBF with chronic use (Kubota et al., 1983), particularly in frontoparietal and occipital regions (Chaarani et al., 2019b; Vafaee et al., 2015), which exacerbates the normative CBF decline observed into late adulthood (Kubota et al., 1983). Consistent with this nicotine-related decline in CBF, we observed greater age and dependence severity-related declines in the CBF of our NTP group compared to our non-using late adolescents. Notably, our sample included primarily e-cigarette/vape users, suggesting consistency in CBF declines across age and nicotine product type.

The identification of the precuneus/PCC and ACC as important regions for nicotine-related alterations of CBF in our model is consistent with evidence suggesting the ACC-precuneus circuit plays a key role in the development of dependence to nicotine (Huang et al., 2014). The precuneus/PCC regions are frequently purported to be involved in nicotine craving and cue-reactivity (Courtney et al., 2014; Engelmann et al., 2012), as is the ACC (Brody et al., 2002; Kühn & Gallinat, 2011; Wilson & Sayette, 2015), particularly in elevated craving states (Culbertson et al., 2011; Hartwell et al., 2011; Wilson & Sayette, 2015). Huang and colleagues (2014) reported a strong negative relationship between withdrawal-induced nicotine craving and white matter tracts connecting the ACC and frontal cortex (including the precuneus), which they speculated might be related to a reduction of top down control over craving networks. Others have reported increased resting state functional connectivity between circuits connecting the ACC, precuneus, and insula during smoking abstinence (Ding & Lee, 2013), further supporting an interconnected role for these regions in nicotine dependence development. Our results add to this body of research by demonstrating a significant negative correlation between dependence severity, and more specifically craving, and CBF in the ACC and PCC. The absence of a relationship between recency of NTP use and CBF suggests this effect is due to chronic NTP use as opposed to acute influences of nicotine.

Limitations of the study include the cross-sectional design which limits our ability to make causal interpretations of the results and the presence of statistically significant group differences in sex and age distributions which may not have been fully accounted for by statistical control. Contrary to the existing literature, we did not observe sex-specific effects in our analyses, and controlling for sex did not alter the reported outcomes. However, we were unable to test the three-way interaction of sex, age, and NTP use group in the whole-brain analysis due to the complexity and limited power of the model. Thus, sex-specific effects may still be present.

## Conclusions

This report represents the first investigation of gray matter health as indexed by CBF in adolescent/young adult users of NTPs. The results support existing literature in adult populations showing age- and nicotine-related declines in CBF in two key regions for nicotine dependence development, namely the ACC and precuneus/PCC. Future prospective investigations of the effects of nicotine on CBF and the roles of pre-existing characteristics are planned for ongoing longitudinal studies. Greater understanding of the potential implications of nicotine use during this vulnerable developmental period will help elucidate the pathways by which substances modulate the brain during adolescence resulting in a greater propensity for addiction and other neuropsychological outcomes.

## Data Availability

The data that support the findings of this study are available from the corresponding author, JJ, upon reasonable request.
